# Understanding the Competition between Alcohol Formation and Dimerization during Electrochemical Reduction of Aromatic Carbonyl Compounds

**DOI:** 10.1021/jacs.5c10757

**Published:** 2025-10-31

**Authors:** Jonah B. Eisenberg, Kwanpyung Lee, J. R. Schmidt, Kyoung-Shin Choi

**Affiliations:** Department of Chemistry, 5228 University of Wisconsin-Madison, Madison, Wisconsin 53706, United States

## Abstract

The electrochemical reductive dimerization of small aromatic carbonyl compounds derived from lignocellulosic biomass is a crucial C–C coupling reaction for upgrading small molecules to long-chain hydrocarbons, particularly in the synthesis of drop-in sustainable aviation fuels. Although other electrochemical reduction reactions of these reactants (i.e., hydrogenation and hydrogenolysis) have undergone extensive mechanistic investigation, the understanding of dimerization remains relatively underdeveloped. Most importantly, there is a lack of understanding of the selectivity-determining step between dimerization and monomer reduction and critical factors that can affect this step. In this study, we provide a comprehensive mechanistic model to explain the competition between dimerization and monomer reduction of benzaldehyde under various conditions. Our model proposes that the selectivity between dimerization and monomer reduction depends on the competition between desorption of a ketyl radical from the electrode, necessary for dimerization, and further reduction of the ketyl radical to an alcohol on the electrode by proton-coupled electron transfer (PCET). Computationally comparing the adsorption/desorption energy and PCET activation barrier energy is challenging because conventional DFT calculations substantially underestimate the PCET kinetic barriers. In this study, we employed constrained DFT-based configuration interaction (CDFT-CI) to obtain a reliable comparison of these energies. Our mechanistic model was tested and supported by experimental results obtained with four electrodes (Cu, Pb, Bi, graphite), three pH conditions (acidic, neutral, basic), and three potentials. Our study offers a coherent mechanistic foundation that can explain how each of these conditions impacts the desorption and PCET processes and the selectivities for dimerization and alcohol production.

## Graphical abstract

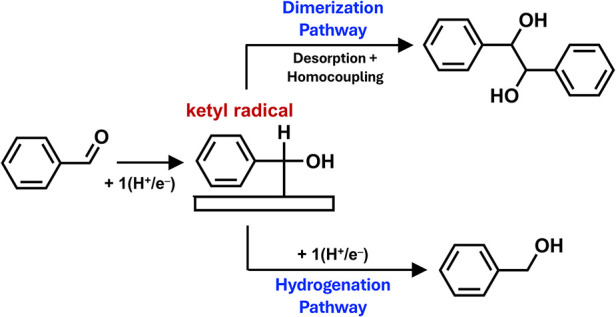

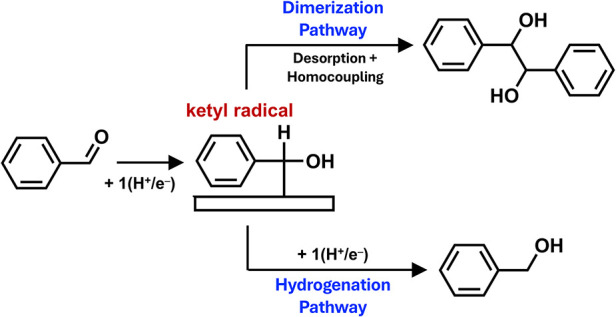

## Introduction

Mitigating carbon emissions requires a sustainable transition from nonrenewable petroleum to renewable plant biomass as a carbon-neutral source of organic compounds and chemical fuels. Lignocellulosic biomass is an attractive carbon feedstock capable of yielding a diverse range of oxygenated aromatic hydrocarbons, many of which contain carbonyl groups (i.e., aldehydes and ketones).^1–3^ These compounds have been upgraded to a myriad of more useful and stable chemicals via reduction or oxidation.^4–6^ The reduction of carbonyl groups can be categorized into three major reactions: hydrogenation, where the carbonyl group is converted to an alcohol; hydrogenolysis, where the C=O bond is broken and the carbonyl group is converted to an alkane; and dimerization, where two carbonyl compounds couple to yield a pinacol dimer (Figure 1a).^6^ Dimerization (i.e., pinacol coupling) is one of many carbon–carbon coupling reactions, which are foundational to organic chemistry and have applications in many industries.^7–10^ In terms of lignocellulosic biomass upgrading, dimerization plays a special role in converting biomass-derived small molecules (C_5_–C_9_) to long-chain molecules, which can be used as drop-in aviation fuels after deoxygenation and hydrogenation.^4,11–15^ In particular, if this reaction is achieved electrochemically, using electricity generated from renewable sources and water as the H source, it can offer a sustainable and environmentally benign route to the production of long-chain hydrocarbons.

**Figure 1:**
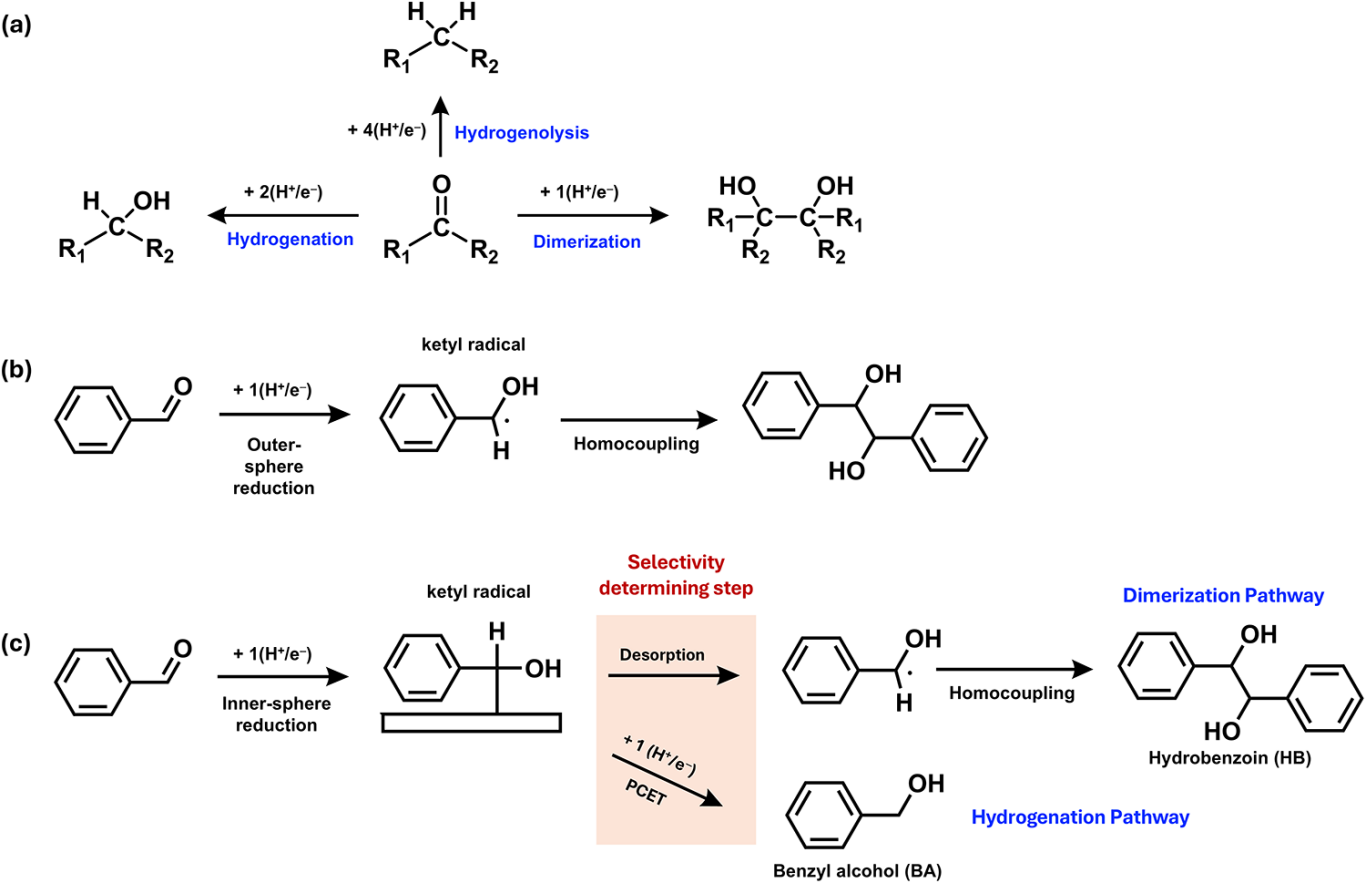
(a) Three possible reduction pathways of a carbonyl compound, (b) a conventional outer-sphere dimerization mechanism for BAL reduction, and (c) a newly proposed mechanism where a ketyl radical forms on the electrode surface and the competition between the desorption of the ketyl radical for radical coupling and further hydrogenation of the ketyl radical on the electrode surface governs the selectivity between dimerization and hydrogenation.

Past studies often treated the dimerization of carbonyl compounds as an undesired side reaction. This is because pinacol products have low demand compared to alcohol and alkane products if they cannot be converted to aviation fuels through further reduction. As a result, dimerization reactions are investigated relatively less intensively, and therefore the current mechanistic understanding of this reaction remains underdeveloped. For example, many papers reported dimerization as an outer-sphere reaction,^16–19^ meaning that the aldehyde reactant is converted to a ketyl radical without adsorbing on the electrode, which is followed by two ketyl radicals coupling to form a pinacol in solution (Figure 1b). However, while pinacol products are almost always obtained in conjunction with other products (i.e., alcohol products), dimerization is often investigated as an independent reaction without specifying the selectivity-determining reaction step. Also, while many papers reported that the pinacol yield is affected by various reduction conditions (e.g., electrode type, potential, pH),^16–32^ how each of these conditions can promote or suppress the dimerization reaction has not been clearly or consistently elucidated. In reality, since dimerization is in competition with other aldehyde reduction reactions, the effect of these variables on dimerization can be explained accurately only when the effects of each of these variables, not only on dimerization, but also on other competing reactions, are comprehensively understood. For example, if one variable increases the yield of a pinacol, it may not be because that variable actively promotes dimerization, but rather because it suppresses the competing reaction pathways.

In the past, our team has extensively investigated the competition between hydrogenation and hydrogenolysis of various aromatic aldehydes (e.g., 5-hydroxymethylfurfural and substituted benzaldehydes).^6,33–39^ From these studies, we established mechanistic models for these pathways which could be used to rationally develop strategies to selectively promote each pathway.^35,36,38^ These studies were performed under the conditions where dimerization is a minor reaction so that the competition between hydrogenation and hydrogenolysis could be more accurately examined without interference from other reactions. From these studies, we showed that hydrogenation is much easier than hydrogenolysis and therefore hydrogenolysis is a minor reaction unless very specific combinations of the reduction conditions are employed.^35,36,38^

In this study, we used benzaldehyde (BAL) as a model compound under the conditions where dimerization and hydrogenation (i.e., benzyl alcohol (BA) production) are the only major reactions in order to gain a comprehensive understanding of their competition through combined experimental and theoretical investigations. Experimentally, we investigated four different electrodes (Cu, Pb, Bi, and graphite) and three different pH conditions (acidic, neutral, and basic) with three different potentials, with an intention to elucidate general trends about the competition between dimerization and hydrogenation observed under widely varying reaction conditions. Computationally, we examined a new hypothesis that may be able to coherently explain all the experimentally observed results from different electrodes (Figure 1c).

In this hypothesis, the first hydrogenation step, yielding a ketyl radical, is the common step between dimerization and hydrogenation. In the low overpotential region, this first hydrogenation step would most likely occur through an inner-sphere reaction, where the resulting ketyl radical is stabilized by the electrode and the degree of stabilization would vary by the nature of the electrode. For dimerization to occur, the desorption of ketyl radicals from the electrode is needed for optimal geometric alignments to form a C–C bond, while BA production requires an additional hydrogenation step on the electrode. *Thus, we hypothesize that the selectivities for dimerization and hydrogenation are determined by the competition between the desorption of a ketyl radical from the electrode vs further reduction of the ketyl radical on the electrode.* This means that comparing the adsorption/desorption energy of the ketyl radical and the activation barrier energy of the ketyl radical reduction to the alcohol product is the key to the computational prediction of reaction selectivity.

We note that computational studies on dimerization have mostly been performed at the level of DFT to date,^18,21^ but conventional DFT is not sufficient to investigate our new hypothesis. This is because although conventional dispersion-corrected DFT works relatively well for adsorption energy calculations, it tends to very substantially underestimate kinetic barriers for the hydrogenation reaction, leading to a strong bias in the comparison of reaction vs desorption barriers.^40^ Hybrid and range-separated DFT functionals, less commonly employed in computational electrocatalysis, offer some incremental improvements, but underestimation of barriers (and thus bias) persists.^41^ Thus, in this study we employed constrained DFT (CDFT)-based configuration interaction (CDFT-CI),^40^ which can more accurately estimate the kinetic barriers for the further hydrogenation of the ketyl radical, which competes with the desorption of the ketyl radical from the electrode. Then, we verified the computational results using the experimental results obtained under various conditions to offer a coherent understanding of the competition between dimerization and hydrogenation pathways during BAL reduction.

## Methods

### Experimental Methods

#### Materials

Benzaldehyde (99+%, Sigma-Aldrich), benzyl alcohol (99.9%, MP Biomedicals), toluene (99.8%, Sigma-Aldrich), *S,S*-hydrobenzoin (98+%, Alfa Aesar), *meso*-hydrobenzoin (99%, Sigma-Aldrich), benzoic acid (99.5%, Sigma-Aldrich), potassium phosphate monobasic (ACS grade, Dot Scientific), potassium phosphate dibasic (≥98%, Sigma-Aldrich), potassium hydroxide (≥85%, Sigma-Aldrich), phosphoric acid (85%, Sigma-Aldrich), potassium sulfate (≥99%, Sigma-Aldrich), boric acid (≥99.5%, Sigma-Aldrich), 2,2,2-trifluoroethanol (99.9%, Oakwood Chemical), sulfuric acid (95.0–98.0%, Sigma-Aldrich), hydrochloric acid (37%, Sigma-Aldrich), acetonitrile (≥99.9%, Sigma-Aldrich), deuterium oxide (99.9 atom % D, Sigma-Aldrich), dimethyl sulfone (98%, Sigma-Aldrich), Cu foil (99.9%, Nimrod Copper), Pb foil (99.9995%, Alfa Aesar), Bi rod (99.99%, Beantown Chemical), and graphite rod (99.999%, Sigma-Aldrich) were used without further purification. All aqueous solutions were prepared using Nanopure water purified from deionized water (Barnstead GenPure Pro Ultrapure water system, resistivity >18 MΩ·cm).

#### Electrode Preparation

All foil electrodes (Cu and Pb) used for organic reduction were prepared by cutting the metals into 1.3 cm × 3 cm strips and masking the front and back with masking tape such that a known surface area was exposed to the electrolyte during experiments. This surface area was 1 cm^2^ for Cu and 2.45 cm^2^ for Pb. The Bi rod was prepared by attaching a piece of Cu tape for electrical contact to one end (top) and masking the top half of the rod with masking tape to expose a surface area of 6.64 cm^2^ on the bottom. Graphite rod electrodes were prepared by masking the middle section with tape such that the top was uncovered for electrical contact to the potentiostat, and the bottom had an exposed surface area of 1 cm^2^. Before use, Bi and Cu electrodes were treated with acid to remove their surface oxide layers. Bi was submerged in 37 wt % HCl for about 10 s. Cu was submerged in 10 wt % H_2_SO_4_ for about 10 s. After the acid wash, each electrode was rinsed with Nanopure water (Barnstead, >18 MΩ·cm) and dried under N_2_ flow. Further removal of surface oxides was performed using either reductive LSVs in blank electrolyte or a constant potential step at moderate reducing potential immediately before benzaldehyde electrolysis at a more negative potential.

#### Electrolyte Preparation

All electrolytes were made using Nanopure water (Barnstead, >18 MΩ·cm). For phosphate-buffered pH 2 and pH 7 electrolytes, an aqueous solution of 0.5 M potassium phosphate was made and adjusted to the desired pH with KOH. Unbuffered pH 13 electrolyte was prepared by dissolving 0.1 M KOH in water. We chose to use 0.1 M KOH (pH 13) instead of 0.5 M phosphate buffer (pH 13) to study the reaction selectivity in alkaline solution because a buffer is not needed in highly alkaline solutions and previous papers dealing with benzaldehyde dimerization in alkaline solutions used 0.1 M KOH (pH 13).^20,30,32^ Thus, providing our data in the same electrolyte gives readers a good reference point to compare our results with previous results. Additionally, since H_3_PO_4_ is a multiprotic acid, the ionic strength of 0.5 M potassium phosphate buffer at pH 13 is considerably higher than those of the corresponding buffers at pH 2 and 7 and 0.1 M KOH. As a result, the use of 0.5 M potassium phosphate buffer at pH 13 can cause additional, unintended changes (e.g., changing the solution conductivity dramatically), which may alter the changes solely caused by the pH. For testing a different proton donor than water at pH 13, a 0.7 M 2,2,2-trifluoroethanol (p*K*_a_ = 12.46) solution was prepared with the pH adjusted to 13 by adding KOH. The concentration of the protonated trifluoroethanol, which can serve as a proton donor, in this solution is expected to be 0.16 M at pH 13.

#### Linear Sweep Voltammetry (LSV)

LSV was performed at room temperature (21 °C) without stirring in a glass H-cell divided by a 3.5 mm thick sintered glass disk. The 3-electrode system consisted of a Cu, Pb, Bi, or graphite working electrode (WE), an Ag/AgCl (4 M KCl) reference electrode (RE), and a Pt foil or Pt mesh counter electrode (CE). The distance between WE and RE was approximately 5 mm. Both cell compartments contained 10 mL of electrolyte and were capped with custom-built Teflon caps to minimize evaporation. A VSP multichannel potentiostat (BioLogic) was used to sweep the potential from the open circuit potential in the negative direction at a scan rate of 50 mV s^–1^.

#### Constant Potential Electrolysis

Constant potential electrolyses (CPEs) were performed at room temperature (21 °C) with stirring, using the same cell setup described in the LSV methods. The anode compartment contained 10 mL of electrolyte, and the cathode compartment contained 10 mL of electrolyte with 10 mM benzaldehyde. Electrolyses consisted of two potential holds: first at a moderate potential to reduce the electrode surface, then at a more negative potential to reduce benzaldehyde. This more negative potential used for benzaldehyde reduction is the applied potential reported in the experimental results section. CPEs were stopped after passing the amount of charge equivalent to 1 electron per reactant molecule (9.649 C for 10 mM BAL) in the second potential step.

#### Product Analysis

Following electrolysis, the solution in the cathode compartment was analyzed using high-performance liquid chromatography (HPLC, Prominence-i LC 2030C 3D, Shimadzu) with a reverse-phase Restek Ultra HPLC column with aqueous C_18_ packing (150 × 4.6 mm, 5 μm inner diameter). The mobile phase was composed of solution A (acetonitrile, 0.1% (v/v) H_3_PO_4_) and solution B (water, 0.1% (v/v) H_3_PO_4_). The flow rate was 1 mL/min, and the column temperature was held at 40 °C. The HPLC gradient began at 10% solution A for 2 min, increased to 45% solution A over a period of 15 min, then increased to 80% solution A over a period of 6 min, and held constant for another 2 min. Concentrations were quantified by comparing analyte peak areas to calibration curves made using product standards. The *dl* and *meso* diastereomers of hydrobenzoin were well-separated on HPLC; as they were generally produced in equal amounts, the concentrations were combined and reported as total hydrobenzoin concentration in the text. Absolute selectivity, Faradaic efficiency (FE), and relative selectivity were calculated using the following equations:

1 $$\mathrm{Absolute Selectivity}\;\left(\%\right)=\frac{\mathrm{mol of BAL converted to specific product}}{\mathrm{mol of consumed BAL}}\times 100$$

2 $$\mathrm{FE}\left(\%\right)=\frac{{\mathrm{mol of e}}^{-}\mathrm{consumed to produce specific product}}{{\mathrm{mol of e}}^{-}\mathrm{passed}}\times 100$$

3 $$\mathrm{Relative Selectivity}\;\left(\%\right)=\frac{\mathrm{mol of BAL converted to specific product}}{\mathrm{total mol of BAL converted to identified products}}\times 100$$

#### NMR Spectroscopy

^1^H NMR spectra were collected on a Bruker Avance III 400 MHz spectrometer with BBFO probe. In a glass NMR tube, 450 μL of aqueous sample was mixed with 50 μL D_2_O containing 12.5 mM dimethyl sulfone as internal standard. NMR analyses were conducted using the WATERGATE method with excitation sculpting to remove the background water signal.

### Computational Methods

The computational methods used in this work can be divided into two parts: (1) adsorption energy calculations for the reactant and intermediate on four electrode materials (Cu, Pb, Bi, and graphite), and (2) PCET activation barrier calculations at the level of CDFT-CI along the minimum energy path (MEP), which is optimized at the level of DFT.

#### Adsorption Energy Calculations

We used the Vienna Ab-initio Simulation Package (VASP)^42–44^ for all conventional DFT calculations. The interaction with core electrons and nuclei were replaced by the projector augmented wave (PAW) pseudopotentials,^45^ and the exchange-correlation of valence electrons was described by using the Perdew–Burke–Ernzerhof (PBE) functional^46^ along with Grimme’s D3 correction.^47^ A kinetic energy cutoff of 400 eV was used for the planewave (PW) basis set. Solvation effects were described by the implicit solvation model^48,49^ (VASPsol) implemented in VASP.

For adsorption energy calculations, Cu (FCC), Pb (FCC), Bi (*R*3*m* space group), and graphite were used. Slab models were constructed using a 3 × 3 × 1 unit cell of Bi(001) and 4 × 4 × 1 unit cells of Cu(111), Pb(111), and graphite(001). The Brillouin zone was sampled with *k*-points grids of 5 × 5 × 1 (Bi) or 4 × 4 × 1 (other electrodes). The bottom two layers were fixed at their bulk positions. Results with the density-dependent energy correction (dDsC) method^50^ were also included in Table S1, as it is known that the D3 method overestimates the adsorption energy of benzene on coinage metals.^51,52^

#### Activation Barrier Calculations

For optimization of the MEP for reduction of the ketyl intermediate, graphene was used as a model electrode. The proton donor was modeled as a solvated proton, H_7_O_3_^+^, where two explicit water molecules solvate the hydronium ion (H_3_O^+^) and the remaining hydrogen points to the proton acceptor. Note that a solvated proton (H_7_O_3_^+^) has been widely used in PCET studies.^35,36,38,53^ The nudged elastic band (NEB) method^54^ was utilized to obtain the minimum energy path. To calculate the constant potential barrier, we applied the grand-canonical DFT (GC-DFT) calculation so that the potential is kept constant relative to the initial configuration, approximately −1.1 V vs the standard hydrogen electrode (SHE) (equivalent to −0.98 V vs the reversible hydrogen electrode (RHE) at pH 2) as an electron is transferred to the electrode due to an ionization of H_7_O_3_^+^. Additionally, we compared the result from the DFT energy with a *k*-point sampling of the gamma point only against the grand-canonical DFT with a 4 × 4 × 1 *k*-point sampling. This showed that the shift in potential minimally affects the barrier, by less than 0.05 eV, justifying the use of a constant charge barrier in the CDFT calculations. Note that all the carbon atoms of graphene were frozen for efficient CDFT calculations. We confirm that this does not produce any nontrivial change in adsorption energy as CHO and CHOH are physisorbed on the graphite surface.

The CDFT calculations for images of the MEP were performed using CP2K.^55^ As the CDFT calculations in CP2K do not support Brillouin zone sampling, we expanded the system to a larger supercell of 8 × 8 × 1 with 128 carbon atoms for graphene. The plane-wave and Gaussian (GPW) basis sets were truncated with 500 and 50 Ry energy cutoffs, respectively. The molecularly optimized DZVP-MOLOPT-GTH Gaussian basis sets^56^ were used to describe valence electrons, and core electrons and nuclei were replaced with norm-conserving GTH-PBE pseudopotentials.^57^ Likewise, PBE with the D3 dispersion correction was used. Also, the implicit solvation model (SCCS)^58^ implemented in CP2K was employed.

We employed fragment-based constraints to define the constraint target values since the formal number of electrons can be poorly defined.^59^ The system is partitioned into subsystems through a two-step process: (1) the total system was first divided into (i) CHOH (with or without the transferred H) and (ii) the electrode (graphene) together with the proton donor (either H_7_O_3_^+^ or 3H_2_O, depending on whether H has been transferred). (2) Subdivision of (ii) is then conducted into (a) graphene and (b) the proton donor (with or without the transferred H) (Figure S1). We applied four constraints, as consistent with the physical meaning of diabatic states: charge and spin constraints to both the ketyl radical and the proton donor. The Becke weight function for real space partitioning was used with its cell boundaries that were shifted using element covalent radius: 0.76, 0.31, and 0.66 Å for C, H, and O, respectively.^60^ The constrained charge/spin convergence criteria were set to be 0.005*e*. Since the IS diabat involves a characteristic of radicals on both graphene and the reactant, we considered two diabats constructed from two constraints considering spin configurations. That is, a total of four diabatic states were used in the CDFT-CI.

Our CDFT-CI calculation was validated by applying our method to a finite cluster model of this system, replacing the periodic graphene with perylene (C_20_H_12_) (Figure S2). Our CDFT-CI calculations were benchmarked against DLPNO–CCSD­(T)^61,62^ with a def2-TZVPP^63^ basis set, using the ORCA quantum chemistry program package.^64^ For DLPNO–CCSD­(T) calculations, the parameters—the PNO occupation threshold (TCutPNO), the pair cutoff (TCutPairs), and the domain size parameter (TCutMKN)—were set to their default values of 3.3 × 10^–7^, 1.0 × 10^–7^, and 1 × 10^–7^, respectively. CDFT-CI calculations were consistent with DLPNO–CCSD­(T) within 0.1 eV for the configurations we considered. Note that this validation test was done with a total charge of +1 without the implicit solvation.

## Results and Discussion

### Experimental Study

BAL was selected for this study because it has been used as a common model compound in previous dimerization studies;^16,21,26,28–30,32^ therefore, the results obtained in this study can be straightforwardly compared to previous studies. In addition, the commercial availability of its dimerization product (hydrobenzoin, HB) makes the quantification and selectivity analysis of products simple and accurate.

To obtain a comprehensive understanding of factors that affect the selectivity between dimerization and hydrogenation of BAL on electrodes having different characteristics, we investigated four different electrodes: Cu, Pb, Bi, and graphite rod. Cu is an electrode that is known to yield a high hydrogenation selectivity^6,36–38^ and thus can be used as a control electrode to reveal characteristics that limit the dimerization selectivity. Pb and graphite are known to yield a high dimerization selectivity for BAL.^16,26,28,32,65^ Bi has not been previously investigated for BAL reduction, but we show herein that Bi can yield superior dimerization selectivity to Pb.

To examine the general effects of pH on the dimerization and hydrogenation selectivities for BAL, we investigated BAL reduction on each of these electrodes in acidic (pH 2), neutral (pH 7), and basic (pH 13) solutions. Strongly acidic conditions (pH < 2) were not used to avoid substantial loss of FE to the hydrogen evolution reaction (HER). Also, strongly alkaline conditions (pH > 13) were not used to avoid undesired disproportionation of BAL to BA and benzoic acid through the base-catalyzed Cannizzaro reaction.^32,66^ For pH 2 and pH 7 solutions, where pH can readily increase during BAL reduction, a 0.5 M potassium phosphate buffer solution was used. The pH 13 electrolyte was not buffered because the increase of pH above 13 should be negligible considering the relative amount of OH^–^ that can be generated during BAL reduction.

Before we performed constant potential reduction of BAL, we first obtained linear sweep voltammograms (LSVs) using Cu, Pb, Bi, and graphite electrodes in pH 2, 7, and 13 solutions, with and without 10 mM BAL, to determine the appropriate potentials to be used for BAL reduction. The results are plotted against the RHE scale (Figure 2). In these plots, the reduction features observed without BAL are due to HER (appearing as a new reduction wave) or the oxygen reduction reaction (ORR) (appearing as elevated reductive background before the HER onset, most evidently shown for the case of graphite^67^), and the reduction features that additionally appear with the presence of BAL are due to BAL reduction.

**Figure 2:**
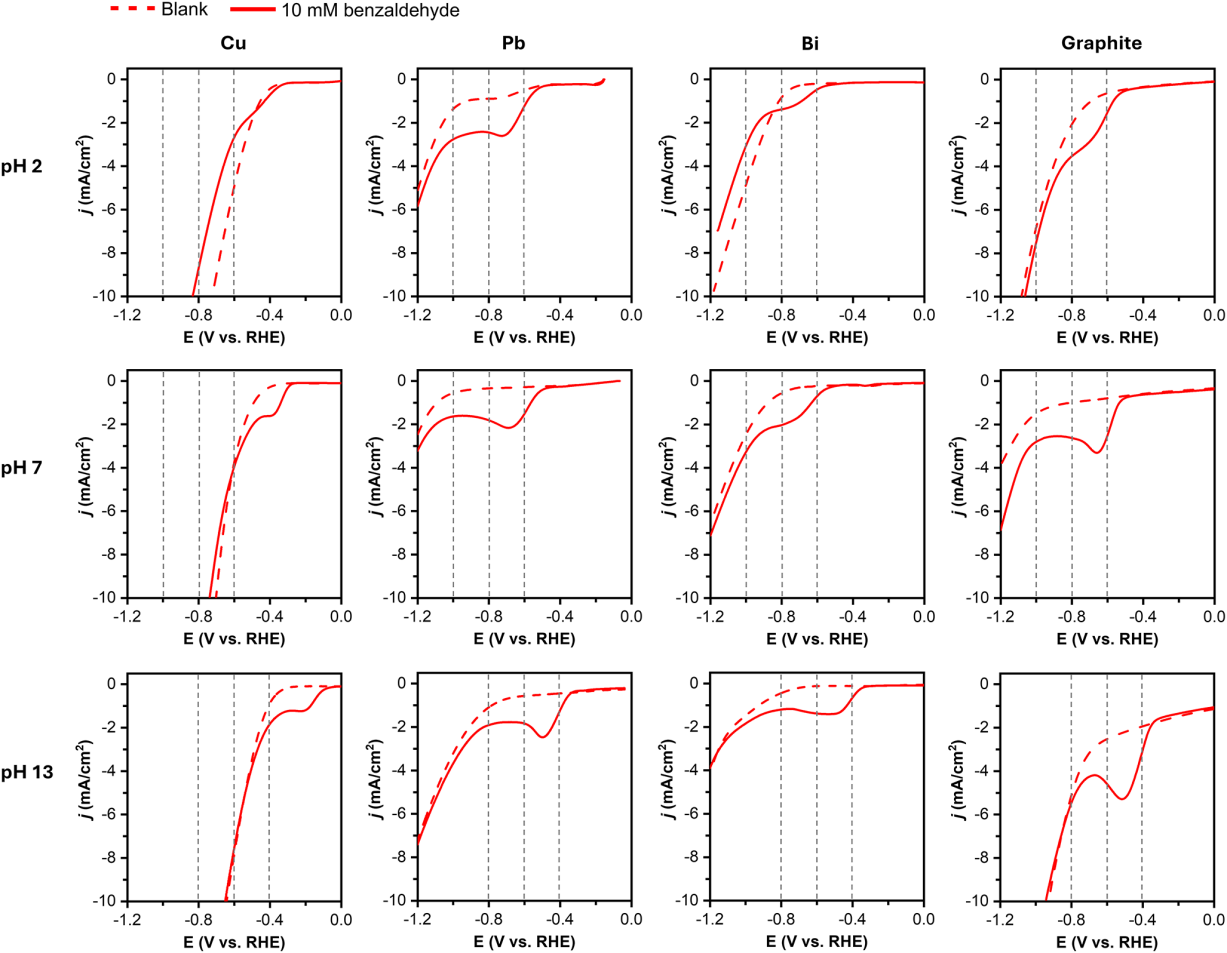
LSV plots of various electrodes in pH 2, pH 7, and pH 13 electrolytes with (solid) and without (dashed) 10 mM BAL (scan rate: 50 mV s^–1^). The vertical dashed lines represent the potentials chosen for the constant potential BAL reduction.

All electrodes show that the onset potential for BAL reduction is less negative than that of HER, meaning BAL reduction is easier than HER in the low overpotential region. The onset for BAL reduction varies by the electrode type (i.e., Cu vs Pb, Bi, and graphite), suggesting that BAL reduction occurs through an inner-sphere reaction. An outer-sphere reaction may become possible in the very negative potential overpotential region. However, the results obtained from constant potential BAL reduction discussed below did not show any indication that an outer-sphere reaction occurred under the potentials examined in this study. (If the outer-sphere reaction is enabled at a more negative potential, an increase in the dimerization selectivity is expected; however, we observed the opposite.) Cu shows the earliest onset for BAL reduction under all pH conditions, meaning it can best stabilize the reaction intermediate, such as the ketyl radical, during the inner-sphere reaction. Although Cu shows the earliest onset for BAL reduction, it is also the best HER catalyst compared to other electrodes used in this study. Thus, the potential window that allows only BAL reduction is narrowest for Cu.

We note that the onset potentials for BAL reduction and HER are determined kinetically and the kinetics of both BAL reduction and HER change by pH; the major proton donors in different pH solutions vary (e.g., H_3_O^+^, H_2_O, conjugate acid of the buffer) and they affect the rate constants for BAL reduction and HER on different electrodes.^68^ Therefore, the onset potentials for BAL reduction and HER were not constant against RHE when pH was varied.

Using the LSV data, we selected three potentials that can be commonly applied to all electrodes at each pH for the constant potential BAL reduction. For pH 2 and pH 7 solutions, we chose −0.6, −0.8, and −1.0 V vs RHE. For pH 13 solution, we chose −0.4, −0.6, and −0.8 V vs RHE. These potentials were chosen to yield sufficient BAL reduction on all electrodes without inducing considerable HER.

We conducted constant potential electrolyses using these potentials. The product distributions and FEs after passing 9.649 C of charge (equivalent to 1 electron per BAL molecule) are shown in Figures 3 and 4. Relative selectivities of identified products are shown in Figure S3. The quantified products were compared to the quantity of consumed BAL, and any discrepancy in the carbon balance is reported as “other.” This discrepancy is caused by some evaporation of BAL, especially for the electrolyses that took longer than others, as well as due to the formation of unidentifiable products (e.g., those formed by oligomerization or acid-catalyzed acetalization).^29,69,70^ All numerical data used to make Figures 3, 4 and S3 as well as electrolysis conditions (average current density and electrolysis time) are summarized in Table S1. Our observations for each electrode material are summarized below:

**Figure 3:**
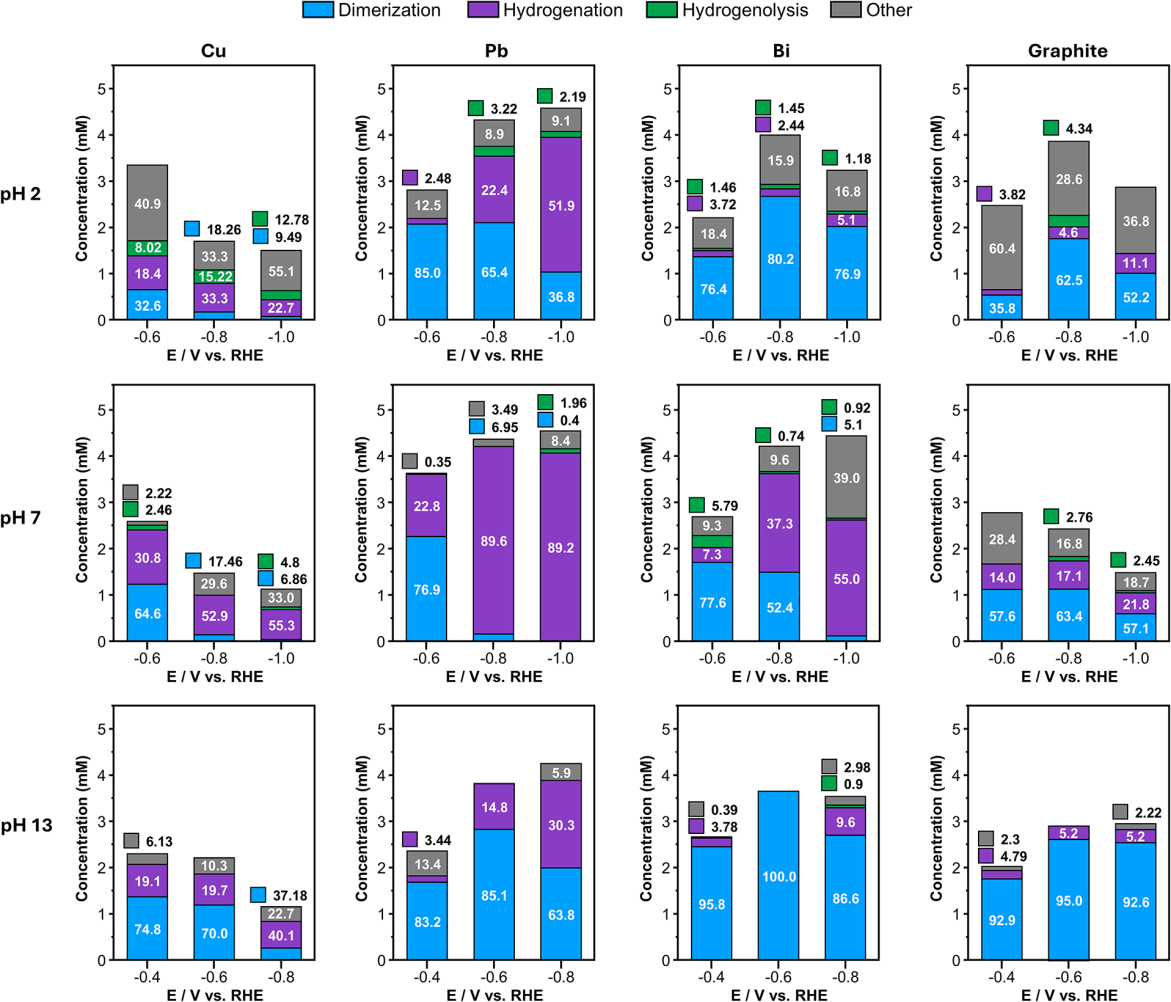
Product concentrations after passing 1 e^–^ per BAL molecule, using Cu, Pb, Bi, and graphite rod electrodes at pH 2, pH 7, and pH 13. Numbers on each bar show the absolute selectivity of each product. Note that two BAL molecules are consumed to form one HB molecule. Thus, the selectivity for dimerization (the number in the bar graph), calculated using the fraction of consumed BAL converted to HB, is different from the relative height in the bar graph, showing the fraction of HB among the products.

**Figure 4:**
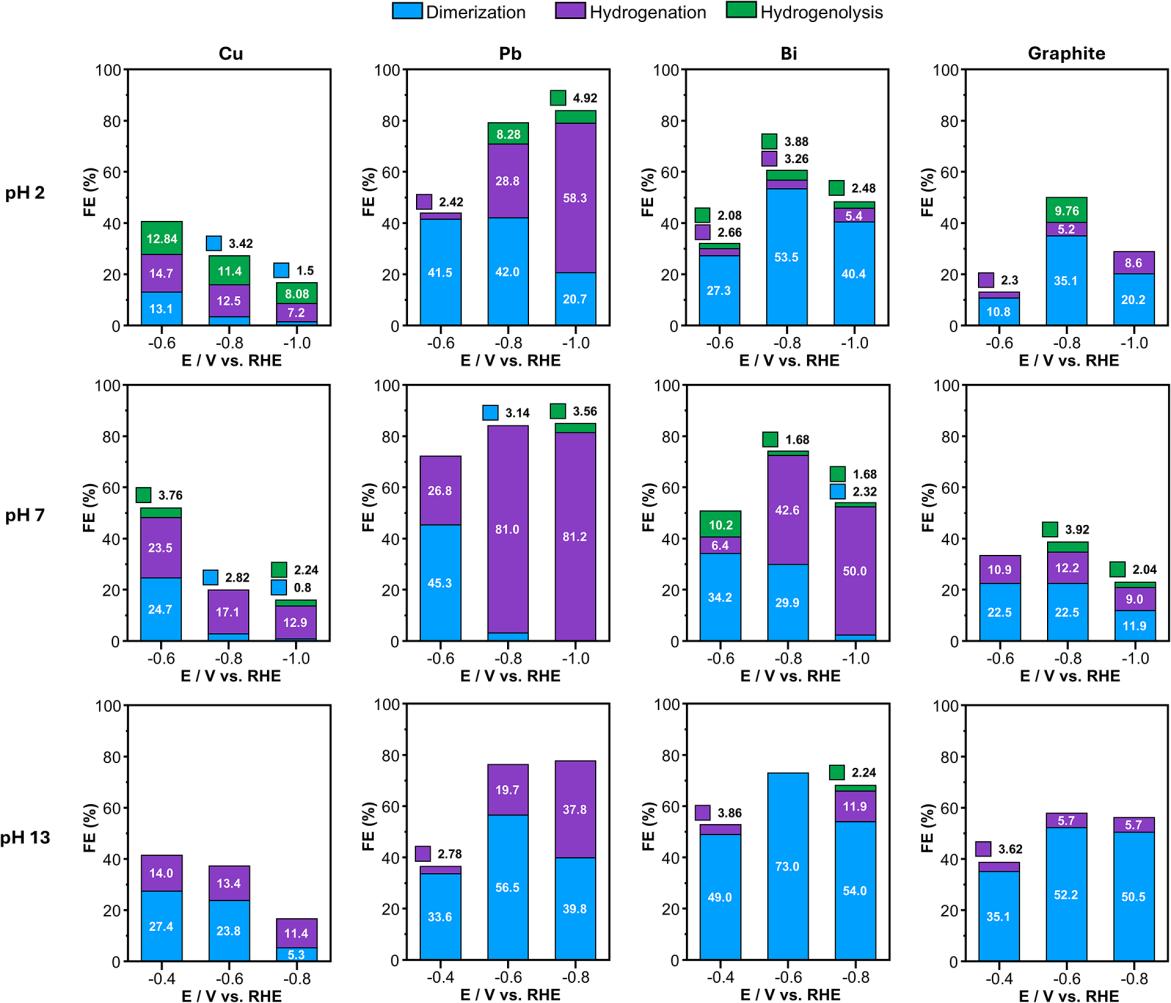
Faradaic efficiencies (FEs) after passing 1 e^–^ per BAL molecule using Cu, Pb, Bi, and graphite rod electrodes at pH 2, pH 7, and pH 13. Numbers on each bar show the numerical value of FE.

**Cu** in general consumed the least amount of BAL compared to other electrodes at all pH conditions, as it has better HER kinetics than other electrodes, thus diverting more current toward HER and away from BAL reduction. Among all electrodes, Cu showed the lowest relative selectivity for dimerization over hydrogenation. At all pH conditions, Cu’s dimerization selectivity decreased as the potential became more negative. Across the pH range investigated, Cu’s hydrogenation selectivity was the highest at pH 7, and Cu’s dimerization selectivity was the highest at pH 13.

**Pb** in general consumed the most BAL compared to other electrodes at all pH conditions as it is the poorest HER electrode among the electrodes used in this study. At any given pH, Pb showed the highest dimerization selectivity at the least negative potential, and its dimerization selectivity decreased as the applied potential became more negative, as in the case of Cu. Across the pH range investigated, Pb showed enhanced hydrogenation at pH 7 and enhanced dimerization at pH 2 and 13.

**Bi** showed the same potential dependencies as Pb, with dimerization selectivity decreasing with more negative potential. Also, Bi showed enhanced hydrogenation at pH 7 and enhanced dimerization at pH 2 and 13 like Pb. Bi generally showed a higher dimerization selectivity than Pb under almost all conditions. In fact, when used at −0.6 V vs RHE in a pH 13 solution, Bi achieved the highest selectivity (100%) and FE (73.0%) for dimerization among all electrodes under any conditions used in this study.

**Graphite** showed dimerization as the dominant reaction under all conditions including pH 7, meaning hydrogenation was most suppressed on this electrode. However, the actual amount of the dimer produced on the graphite was less than those on Pb and Bi because HER and ORR are more substantial on graphite than on Pb and Bi, lowering the FE for dimer production. Graphite showed the same potential and pH dependence for dimerization selectivity as Pb and Bi; the hydrogenation selectivity increased with a more negative potential, and the hydrogenation selectivity was the highest at pH 7 compared to the acidic and basic solutions.

From all experimental results, we identified the following general trends for all electrodes:

- Dimerization is more favored at a less negative potential, while hydrogenation is more favored at a more negative potential.
- When pH increases from 2 to 7, all electrodes show enhanced hydrogenation selectivity at the same potential vs RHE.
- When pH increases from 7 to 13, all electrodes showed enhanced dimerization selectivity at the same potential vs RHE.

In the next section, we discuss a mechanistic model and computational results that can coherently explain the experimentally observed effects of the electrode, pH, and potential on the competition between dimerization and hydrogenation.

### Computational Investigation

#### Adsorption Energy Calculations

We first calculated the adsorption energy of BAL (denoted as CHO, representing the aldehyde group) and a ketyl radical (denoted as CHOH), obtained by adding H to the carbonyl O of BAL, on Cu, Pb, Bi, and graphite (Figure 5). In our previous study, we showed that the barrier to initial PCET at the carbonyl carbon is significantly higher than that at the carbonyl oxygen, and it is safe to assume that the carbonyl reduction involves the formation of a ketyl radical as the first step.^38^ Also, the formation of the dimer product through C–C coupling provides an additional experimental confirmation that the initial PCET results in the formation of a ketyl radical.

**Figure 5:**
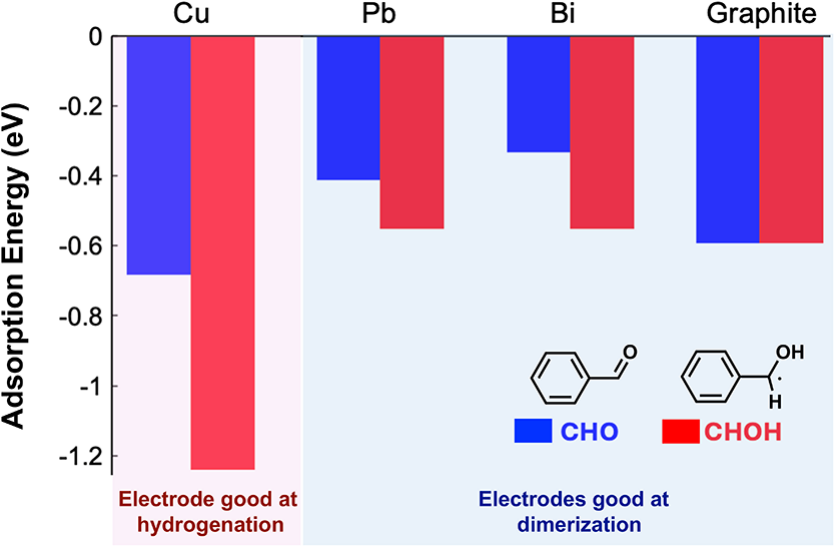
Adsorption energies (in eV) of CHO (blue) and CHOH (red) on Cu, Pb, Bi, and graphite. Clean slabs of the electrodes and isolated CHO and CHOH are used as references. The density-dependent energy correction (dDsC) was used instead of the D3 method. The values using the D3 method can be found in Table S2.

Figure 5 shows that both CHO and CHOH have negative adsorption energies, meaning they are more stable on the electrode than in solution. In general, the CHOH intermediate is more strongly adsorbed than the CHO reactant, suggesting that the ketyl radical is stabilized by the presence of the electrode surface. The stabilization of the radical intermediate increases the driving force for the first reduction step, thus reducing the overpotential. Indeed, the stabilization of these species is the largest on Cu, which exhibits the least negative onset potential for BAL reduction (Figure 2). These results, as well as the experimentally observed fact that the onset potentials for BAL reduction on Cu, Pb, Bi, and graphite electrodes are not the same, suggest that the reduction of CHO to CHOH occurs through an inner-sphere reaction, at least in the low overpotential region near the onset of BAL reduction.

We hypothesize that for surface-adsorbed ketyl radicals to undergo dimerization, the desorption of the radicals is needed to optimally configure two radicals to form a C–C bond. This dimerization step is not a reduction step and is expected to be (mostly) potential-*independent*. Thus, the energy needed for desorption of the ketyl radical (i.e., desorption barrier energy, equivalent to the adsorption energy of CHOH on the electrode) is expected to be a key factor in determining how easily dimerization can occur. On the other hand, the further reduction of the ketyl radical, which occurs in competition with desorption, depends on the kinetic barrier height for the corresponding proton-coupled electron transfer (PCET) step. This barrier height is potential-*dependent*, decreasing with increasing overpotential (i.e., a more negative applied potential). We believe that ketyl radical reduction by hydrogen atom transfer (HAT) of the surface adsorbed H from the electrode can be ruled out in this discussion, as the hydrogen coverages are expected to be negligible given that the HER current near the onset potential for dimerization is minimal on Pb, Bi, and graphite (Figure 2).

The above hypotheses are consistent with the experimental observation that the dimerization selectivity, which competes with the hydrogenation selectivity, decreases with increasing overpotential. We also note that Cu, with the strongest adsorption for both CHO and CHOH, showed the lowest dimerization selectivity compared to the other electrodes, suggesting the importance of the adsorption energy in affecting the competition between dimerization and hydrogenation.

Based on these observations, we built a mechanistic model where the competition between dimerization and hydrogenation is governed by the desorption barrier of the ketyl radical from the electrode and the PCET barrier for the ketyl radical reduction on the electrode (Figure 1c). To verify this mechanistic model, we next calculated the kinetic barriers for the reduction of the ketyl radical and examined if our calculation results can explain the experimentally observed selectivity trends. We note that the reduction of CHO* to CHOH* is a common step for both dimerization and hydrogenation and thus does not directly affect the selectivity. We therefore examined the PCET barriers of only the second PCET step of hydrogenation, where a ketyl radical is reduced to an alcohol.

#### PCET Barrier Calculations

As briefly mentioned in the introduction, standard DFT tends to underestimate kinetic barriers, whereas thermodynamic properties, like adsorption energy, can be relatively well captured.^41^ This systematic underestimate arises, in part, due to DFT delocalization and self-interaction errors, exacerbated by the multireference character that is often present during bond breaking/formation. Here, we utilize constrained DFT (CDFT)-based configuration interaction (CI), CDFT-CI, to yield substantially more accurate estimates of the PCET barriers.^40^

In CDFT, charge or spin constraint potentials are added to the Kohn–Sham Hamiltonian. The ground state solutions of this constrained Hamiltonian yield effective diabatic states, where, for example, a transferring electron has been constrained to reside on a donor or acceptor moiety instead of possibly being delocalized between them. In contrast to the unconstrained (adiabatic) ground state obtained from DFT, these constrained diabatic states typically exhibit far fewer self-interaction errors. Using the various diabatic states (differing, for example, in the location of the electron) in conjunction with calculated couplings between the diabats, CDFT-CI yields an improved adiabatic ground state, with associated activation barriers, of accuracy that typically far exceeds conventional (unconstrained) DFT.^59^

CDFT-CI has previously been applied to electrochemical PCET, examining the Volmer step of the hydrogen evolution reaction.^71^ Here, we examine a more complicated example of PCET to an adsorbed reactant molecule bound to an electrode surface. This requires a careful consideration of the CDFT diabatization scheme, illustrated in Figure 6. We partition the system into three subsystems: reactant (CHOH*), proton donor (solvated hydronium ion), and electron donor (electrode). Each diabatic state corresponds to a localized electron and proton on a particular subsystem, with the electron localization enforced via CDFT, where the proton is included in either the proton donor (pre-PT) or the reactant (post-PT) subsystem. This yields five key diabatic states: the initial state (IS) with both the proton and electron localized on their respective donors; an ET state with only the electron transferred to the reactant; a PT state with only the proton transferred to the reactant; a FS with both the electron and proton transferred to the reactant; and a HAT state with the electron transferred to the proton. Our preliminary calculations show that diabats other than IS, FS, and ET (i.e., PT and HAT diabatic states) have a negligible effect on the ground state energies. Therefore, we excluded them from ground state energy calculations for efficiency.

**Figure 6:**
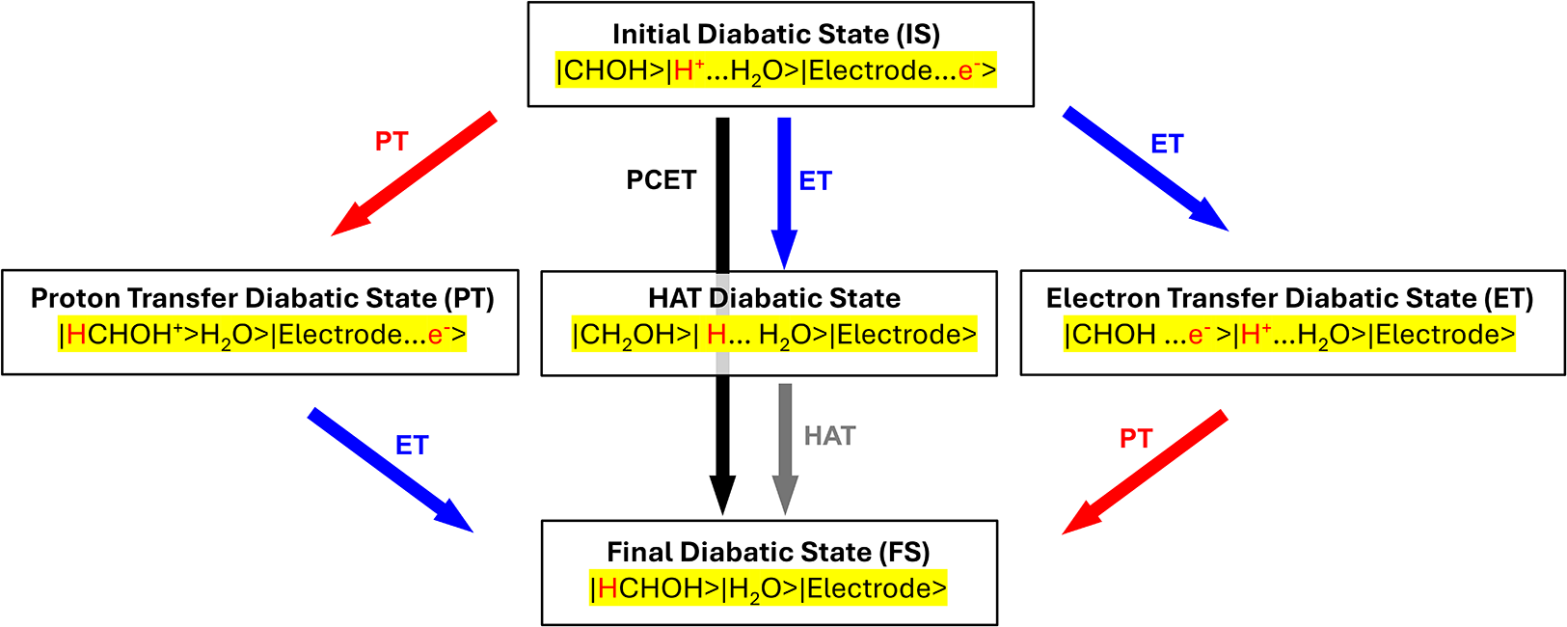
Diabatic states for an electrochemical PCET of CHOH to CH_2_OH. Each diabatic state consists of three subsystems: CHOH reactant, H_2_O, and electrode, where e^–^ (or H^+^) denotes the localization of a transferring electron (or proton) onto a given subsystem. The FS diabat contains an H in the reactant as the result of both PT and ET to the reactant.

Herein, we used graphene (e.g., a monolayer of graphite) as a model electrode for our CDFT calculation due to the current implementation of CDFT in CP2K built on the orbital transformation, which is inappropriate for metallic systems. However, given the similarity in adsorption energies of CHO and CHOH on Pb, Bi, and graphite electrodes (Figure 5), we believe our insight from calculations with graphene can be transferrable to the case of Pb or Bi. We also validated our CDFT-CI calculated PCET barriers against a high-level coupled cluster benchmark (DLPNO–CCSD­(T)), using a smaller graphene fragment (i.e., perylene, C_20_H_12_) as a model electrode. The result (Figure S2) shows that the CDFT-CI calculated PCET barriers reproduce the benchmark values to within ∼0.1 eV, dramatically better than either pure GGA (PBE) or hybrid (PBE0) functionals.

In our calculations, we defined the relevant PCET reaction coordinate as the distance between the transferring proton and the proton donor (i.e., O in H_2_O). We then examined the energetics of the various diabatic states at various positions along the reaction coordinate, using reaction geometries obtained from nudged elastic band (NEB) calculation at the DFT level (Figure 7a). As expected, we find that the IS diabat is more stable than other diabatic states for very small values of the reaction coordinates (near the initial configuration), while the FS diabat becomes more stable for those near the final configuration. More interestingly, the ET diabat exhibits nearly constant energy along the reaction coordinate, and it is more stable than the FS diabat until the reaction coordinate of ∼1.2 Å (just after the transition state at ∼1.1 Å).

**Figure 7:**
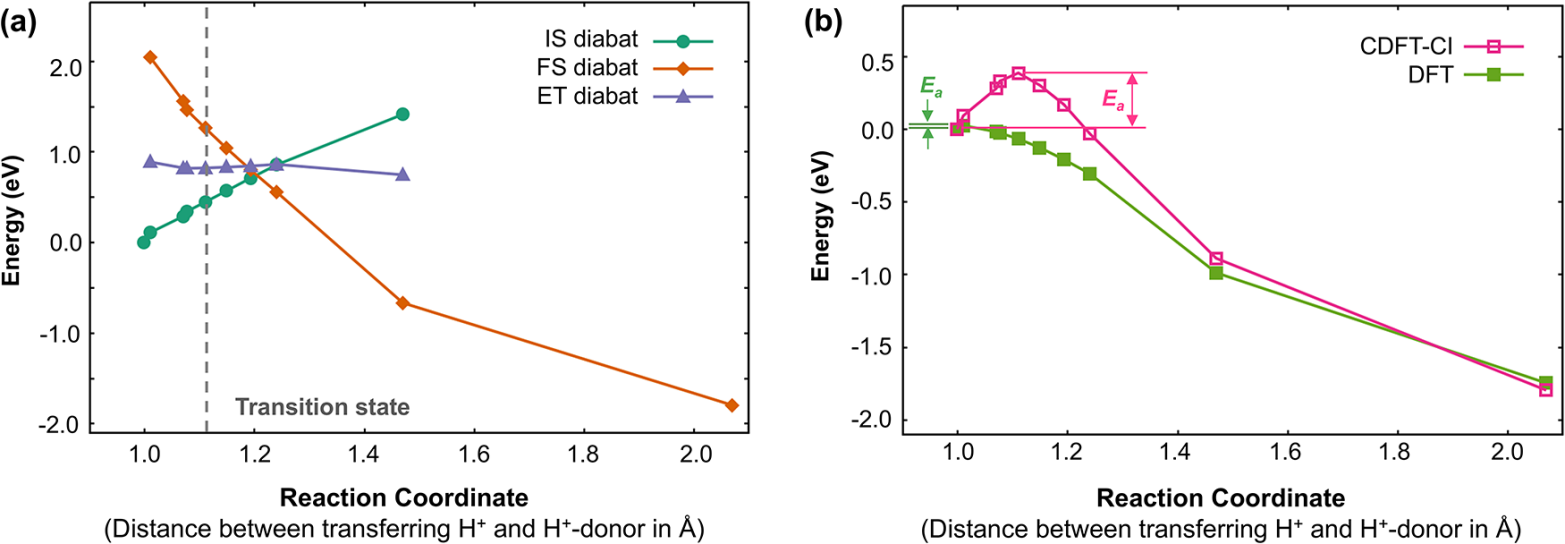
(a) The energetics of IS, FS, and ET diabats along the reaction coordinate during the reduction of a ketyl radical. The reaction coordinate corresponding to the transition state is denoted as a gray dashed line. (b) The potential energy curve during the reduction of a ketyl radical. The PCET activation barrier is denoted as *E*_a_. An electrode potential of −1.1 V vs SHE was applied at the initial configuration.

Building on the CDFT calculations, we performed CDFT-CI calculations to determine the adiabatic ground state energies along the reaction coordinate and compared it with the adiabatic ground state obtained from standard DFT (Figure 7b). The PCET activation energy barrier, *E*_a_, is the difference between the energy at the initial state and the highest energy along the reaction coordinate. The results show that the barrier predicted by the standard DFT is less than 0.1 eV while that predicted by CDFT is 0.39 eV. This discrepancy clearly shows the underestimation of the barrier height by standard DFT due to delocalization error and multireference character, particularly during bond formation.

The PCET activation *free energy* barrier predicted by CDFT-CI is greater when considering the entropic contributions. For example, the entropic contributions for PCET reactions were estimated to be around 0.2 to 0.3 eV for the case of Pt/water interfaces.^72^ We expect that the entropic contributions in the case of graphene/water interfaces would be slightly lower, considering the weaker adsorption of water molecules on graphite than Pt, which would decrease the equilibration time scale of interfacial water molecules.^73,74^ On the other hand, the desorption free energy barrier of the ketyl radical on graphite will *decrease* when considering the entropic contributions. The loss in translational entropy upon adsorption is ∼0.1 eV for chemisorption (gained during desorption), which was approximated as the cratic entropy.^75^ Overall, including the entropic contributions, we estimate a PCET free energy barrier of ∼0.6 eV (0.39 eV + 0.2 eV) vs a desorption barrier of ∼0.4 eV (0.53 eV–0.1 eV), implying that further reduction of the ketyl radical is less feasible than the desorption of the ketyl radical, thus explaining the high selectivity for dimerization on graphite. The electrode potential used in the above PCET calculations was −1.1 V vs SHE (equivalent to −0.98 V vs RHE at pH 2). Thus, when a less negative potential is used, the PCET barrier will increase, further favoring desorption/dimerization against competing hydrogenation. It is important to note that if we used the (underestimated) PCET barrier obtained from conventional DFT, the PCET barrier would be erroneously predicted to be *lower* than the desorption barrier even after entropic considerations, which would fail to offer insights into the experimentally observed high dimerization selectivity on graphite.

CDFT-CI also provides a conceptual understanding of how the electronic structure evolves along the reaction coordinate, providing deeper insights into the details of the PCET process. Our calculations show that near the initial configuration, the weight of the IS diabat is >0.99, meaning the adiabatic ground state can be described (unsurprisingly) solely by the IS diabat. Along the reaction coordinate, as the proton moves from donor to acceptor, the IS diabat becomes less stable and the contributions from the ET and FS diabats increase. Interestingly, the contribution of the ET diabat to the adiabatic ground state is greater than the FS diabat at configurations before and at the transition state. This is because the ET diabat is more stable than the FS diabat at these configurations, as shown in Figure 7a. Specifically, at the transition state, our calculations show that the weight of the ET diabat is 0.54 while that of the FS diabat is 0.36; the contribution of each state along the reaction coordinate can be found in the Supporting Information (Figure S4). As such, roughly speaking, one can view this adiabatic PCET reaction as one that involves substantial electron transfer character early along the reaction coordinate, followed by proton transfer as the proton moves along the remainder of the reaction coordinate—*with the kinetic PCET barrier dictated in large part by the relative stability of the ET diabat*. That said, it is important to emphasize that neither pure electron transfer states (in absence of proton motion) nor proton transfer states (in absence of electron transfer) are stable intermediates, and thus the process remains as concerted PCET. Note that since the ET diabat is expected to further decrease in energy with more negative applied potential, the ET state would be expected to eventually become a stable intermediate when a very negative potential is applied. This would create the possibility for sequential ET/PT (in addition to concerted PCET) at potentials substantially more reducing than the onset potential for BAL reduction.

### Combining Experimental and Computational Results

After establishing that the relative selectivity for dimerization over hydrogenation is determined by the desorption barrier relative to the PCET barrier for the second reduction step, the experimentally observed trends can be explained coherently.

#### The Effect of the Potential on Selectivity

One of the general trends observed for all electrodes is that dimerization is enhanced at a less negative potential, while hydrogenation is enhanced at a more negative potential. This is because the potential-dependent PCET barrier decreases as a more negative potential is applied. As a result, the relative rate of hydrogenation increases, thus increasing the relative selectivity for hydrogenation.

#### The Effect of pH on Selectivity

When pH increased from 2 to 7, all electrodes showed enhanced hydrogenation selectivity at the same potential vs RHE. For the same potential vs RHE, the applied potential against the SHE becomes more negative at higher pH, which decreases the PCET barrier according to the charge transfer coefficient.^76^ (Note that the PCET barrier is related to the rate constant and not the rate. Therefore, the PCET barrier is independent of the proton concentration for a given proton donor, and it changes with V vs SHE and not V vs RHE.) This effect appears to outweigh other changes resulting from the pH increase, thereby enhancing the hydrogenation selectivity.

When pH increased further from 7 to 13, however, all electrodes showed decreased hydrogenation selectivity at the same potential vs RHE, although the applied potential vs SHE became even more negative. This can be explained by considering certain factors that also change with pH to increase the PCET barrier or decrease the desorption barrier, which would increase the relative rate of dimerization over hydrogenation. First, it is important to note that the adsorption (and thus desorption) energy of the ketyl radical itself exhibits a weak potential dependence, slightly weakening with more negative applied potential.^35^ This effect depends on the *absolute* applied potential (i.e., on the SHE scale) and is therefore enhanced at the same potential vs RHE at pH 13. If the resulting decrease in the desorption barrier of the ketyl radical at pH 13 is non-negligible, it would result in an increase in the relative selectivity for dimerization over hydrogenation at pH 13. (If the ketyl radical became deprotonated at pH 13, the desorption barrier would decrease further, as discussed below.)

Second, as pH changes, the proton donor that supplies protons for PCET also changes, which can affect the rate of PCET. We note that at pH 2, pH 7, and pH 13, the major proton donor for PCET can differ:^77^ H_3_O^+^ at pH 2, a conjugate acid of the buffer (H_2_PO_4_^–^) at pH 7, and H_2_O at pH 13. Judging from the p*K*_a_ values of the different proton donors, their proton donating ability is in the order of H_3_O^+^ (p*K*_a_ = 0) > H_2_PO_4_^–^ (p*K*_a_ = 7.2) > H_2_O (p*K*_a_ = 14). It appears that while H_2_PO_4_^–^ at pH 7 is still a good enough proton donor, the extremely poor proton donating ability of H_2_O at pH 13 becomes a limiting factor for PCET, even when a significantly more negative potential vs SHE is applied. Then the rate of the second hydrogenation step would decrease, thus increasing the dimerization selectivity.

In order to verify our claim that the proton donor can influence the hydrogenation selectivity, we performed a control experiment where we added trifluoroethanol as a proton donor in a pH 13 solution, while other reduction conditions were kept identical. Trifluoroethanol (p*K*_a_ = 12.46)^78^ is a slightly better proton donor than H_2_O, although any acid that can serve as a proton donor at pH 13 is a poor proton donor. Our results show a meaningful increase in the hydrogenation selectivity with trifluoroethanol for all electrodes that favor dimerization at pH 13 (Pb, Bi, graphite) (Figures S5–S6), supporting that the poor proton donating ability of H_2_O is one of the factors that decrease the hydrogenation selectivity at pH 13.

In addition, it may even be possible that at pH 13, due to the combination of a very negative potential vs SHE facilitating electron transfer and H_2_O slowing down the proton transfer, sequential ET followed by PT may occur instead of PCET. This means that the first hydrogenation step, which was assumed to be the common first step for hydrogenation and dimerization in Figure 1c, may no longer be the common step. Rather, in this alternative pathway, only the first ET step would be the common step (Figure 8). Subsequently, the initial ET product (the ketyl radical anion) may desorb from the electrode prior to the first PT, and undergo subsequent steps needed for dimerization in solution (Figure 8a). In this case, the current for dimerization, which is affected only by ET, would not decrease at pH 13. In contrast, the current for hydrogenation will decrease because the first PT must be completed with H_2_O serving as a poor proton donor before the second PCET or ET occurs, thus decreasing the hydrogenation selectivity (Figure 8b).

**Figure 8:**
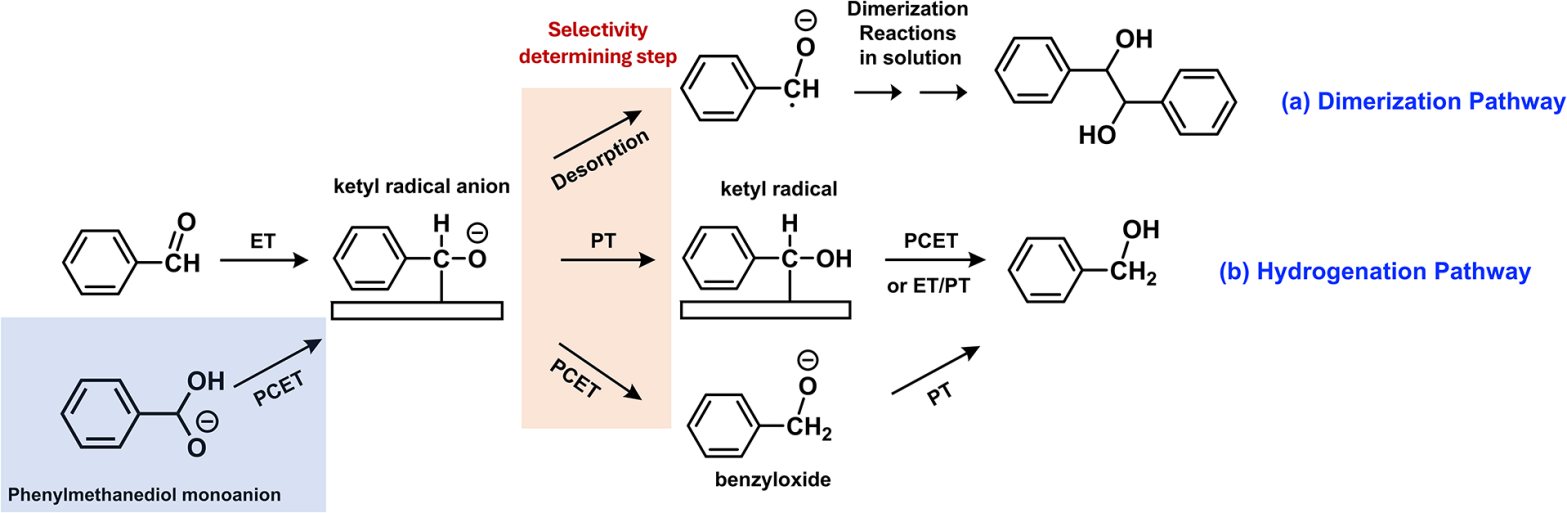
Plausible alternative reaction pathways for (a) dimerization and (b) hydrogenation at pH 13. The blue box shows that even if geminal diol reduction occurs, it would result in the formation of the same ketyl radical anion, not affecting the selectivity determining step.

We also note that the p*K*_a_ of the ketyl radical in ethanol was reported to be 8.14.^79^ If we assume that the p*K*_a_ of the ketyl radical in aqueous solution is similar, the ketyl radical would deprotonate and be present as the ketyl radical anion at pH 13. This consideration increases the chance that the first hydrogenation step at pH 13 would initiate by ET rather than the concerted PCET. The p*K*_a_ of the ketyl radical also suggests that after the first ET, the first PT may not occur to the ketyl radical anion by itself at pH 13 (the upper route in Figure 8b). Considering that the second ET is unlikely to occur until the ketyl radical anion is converted to a neutral ketyl radical by the first PT, it may be possible that the first PT and the second ET may occur in a concerted manner at pH 13, resulting in a benzyloxide intermediate that will easily undergo PT in pH 13 due to the higher p*K*_a_ (15.4) of its conjugate acid, BA (the bottom route in Figure 8b).^80^

The formation of the ketyl radical anion at pH 13 can also affect the desorption barrier. The applied potentials vs SHE used at pH 13 (<−1.17 V vs SHE) are much more negative than the point of zero charge (PZC) of the electrodes used in this study (e.g., ranging between −0.62 V vs SHE for Pb and 0.10 V vs SHE for graphene).^81,82^ Thus, the ketyl radical anion formed at pH 13 would be less stabilized than the neutral ketyl radical on the negatively charged electrode surface, increasing its propensity to desorb and thus further increasing the selectivity for dimerization.

We also considered the fact that about 65% of BAL is present in pH 13 as its germinal diol form, phenylmethanediol.^83,84^ The p*K*_a_ of phenylmethanediol has been estimated to be ∼10,^84^ which means that at least one diol group would be deprotonated at pH 13, resulting in the formation of an anion. We do not think this anionic species can easily be adsorbed on the negatively charged electrode surface to undergo the first reduction step. Thus, we think the aldehyde is the preferred reactant over the geminal diol at pH 13. Once aldehyde is consumed, the fast equilibrium between aldehyde and geminal diol (Figure S7) will replenish the aldehyde, allowing for continuous aldehyde reduction. Even if the geminal diol ever undergoes the first reduction step (see the blue box in Figure 8), the resulting intermediate is the same as that formed by the first reduction step of aldehyde, the ketyl radical anion. Since the selectivity between alcohol production and dimerization depends on the kinetic barriers for the second reduction step and the desorption barrier of the ketyl radical anion, the selectivity would not be affected whether the species that undergoes the first reduction step is aldehyde or geminal diol.

Regardless of various possible mechanistic changes for hydrogenation at pH 13, it is clear that the relative current for hydrogenation over dimerization will decrease due to the difficulty for PT at pH 13, considering the acidity of the ketyl radical, the poor proton donating ability of water, and the destabilization of the ketyl radical or ketyl radical anion on the electrode.

We note that a quantitative discussion on the PCET kinetic barrier depending on the proton donor requires more complicated comprehensive work. However, the above qualitative discussion, considering different proton donors and the effect of pH at constant potential against RHE, offers invaluable insights for understanding the experimentally observed results.

## Conclusion

We combined experimental and computational investigations to develop and verify a mechanistic model that can comprehensively explain the competition between dimerization and hydrogenation during the electrochemical reduction of BAL under various conditions. Experimentally, we conducted BAL reduction on Cu, Pb, Bi, and graphite electrodes in pH 2, 7, and 13 solutions using three different potentials. Cu showed the lowest dimerization selectivity, while Pb, Bi and graphite commonly showed a high dimerization selectivity. The dimerization selectivity on all electrodes decreased with a more negative potential at all pH conditions. When the pH increased from 2 to 7, the relative hydrogenation selectivity increased on all electrodes at the same potential vs RHE. When the pH increased further from 7 to 13, the relative dimerization selectivity increased on all electrodes at the same potential vs RHE. Our mechanistic model proposes that the selectivity between dimerization and alcohol production is determined by the competition between the desorption of the ketyl radical from the electrode and further reduction of the ketyl radical on the electrode, after the ketyl radical is first formed through an inner-sphere reduction reaction. In order to explain the experimental results, we calculated the adsorption/desorption energy of the ketyl radical on Cu, Pb, Bi, and graphite and the PCET activation energy for the ketyl radical reduction on graphite using CDFT-CI. Our results showed that Cu, which showed the lowest dimerization selectivity, has the highest desorption barrier for the ketyl radical, while Pb, Bi, and graphite, which showed a high dimerization selectivity, commonly have a low desorption barrier. Our computational results also showed that on graphite the PCET barrier of the ketyl radical reduction is higher than the desorption barrier of the ketyl radical, explaining the high dimerization selectivity on graphite. Our model explained that the decrease in the dimerization selectivity with a more negative potential on all electrodes is due to the more negative potential decreasing the PCET barrier, thus increasing the relative rate for hydrogenation over dimerization. The increase in the hydrogenation selectivity at the same potential vs RHE when pH increases from 2 to 7 is a result of the applied potential vs SHE becoming more negative with increasing pH, decreasing the PCET barrier, thus increasing the rate of hydrogenation. The increase in the dimerization selectivity at the same potential vs RHE when pH increases from 7 to 13 is due to multiple factors that increase the dimerization rate and decrease the hydrogenation rate at pH 13. These factors include a decrease in the desorption barrier of the ketyl radical or ketyl radical anion, H_2_O serving as a poor proton donor at pH 13, and the possibility of aldehyde reduction occurring via ET followed by PT instead of concerted PCET.

## Supplementary Material



## Notes

Relative selectivities of products, additional computational methods and results, and LSV and product comparison at pH 13 with and without trifluoroethanol as an additional proton donor, NMR of BAL at various pH conditions, tables showing the detailed BAL reduction results, adsorption energies of CHO and CHOH on various electrodes (PDF)
