# Supplementary material for: Understanding the Competition between Alcohol Formation and Dimerization during Electrochemical Reduction of Aromatic Carbonyl Compounds
Source: J Am Chem Soc. 2025 Oct 31;147(45):41390–403. doi: 10.1021/jacs.5c10757 (PMC12616691; doi:10.1021/jacs.5c10757)
Supplement: Supplementary file 1 [file ja5c10757_si_001.pdf]

Supporting Information

Understanding the Competition between Alcohol Formation and Dimerization during Electrochemical Reduction of Aromatic Carbonyl Compounds

Jonah B. Eisenberg,+[a] Kwanpyung Lee,+[a] J. R. Schmidt,*[a] and Kyoung-Shin Choi*[a]

[+] These authors contributed equally to this work.

[a] Department of Chemistry, University of Wisconsin-Madison, Madison, WI 53706, USA

[*] Email: schmidt@chem.wisc.edu, kschoi@chem.wisc.edu

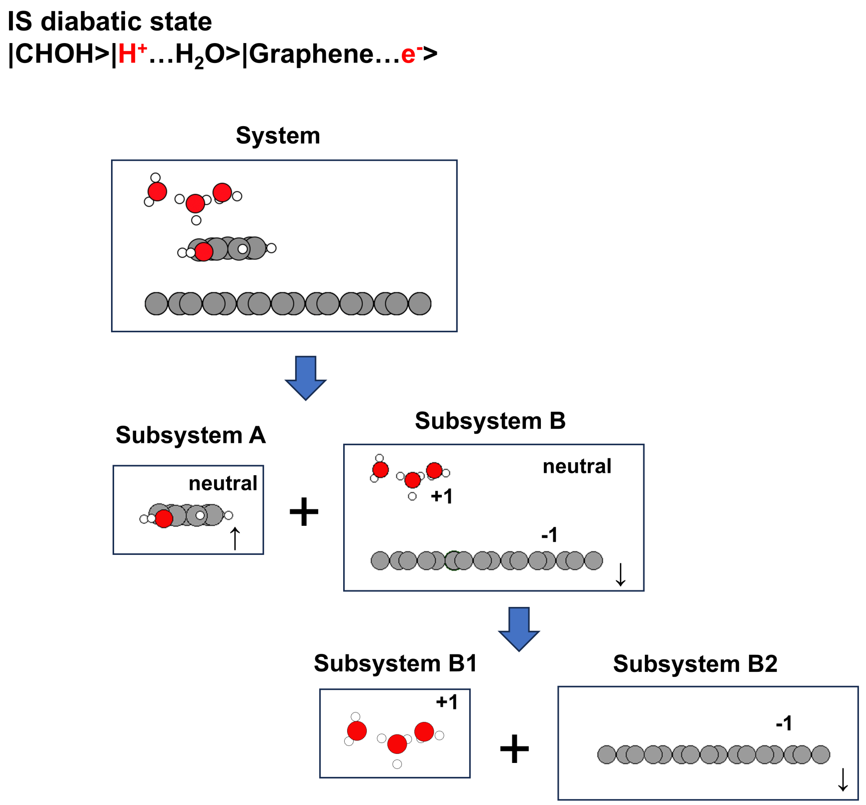

Figure S1. A schematic figure for the process of defining constraints, taking the initial state as an example. Note that the program used in this study can partition the total system into only two subsystems. Thus, we initiated calculations using Subsystem A and Subsystem B: Subsystem A contains the reactant (ketyl radical) and Subsystem B contains the electrode with one electron (the electron from H) and $H_7O_3^+$ (H_3O^+ solvated by two H_2O molecules). Then Subsystem B was further divided into Subsystem B1 and Subsystem B2: Subsystem B1 contains $H_7O_3^+$ and Subsystem B2 contains the electrode plus one electron. The arrows in the figure denote the spin states of electrons in cases where the subsystem is not a singlet.

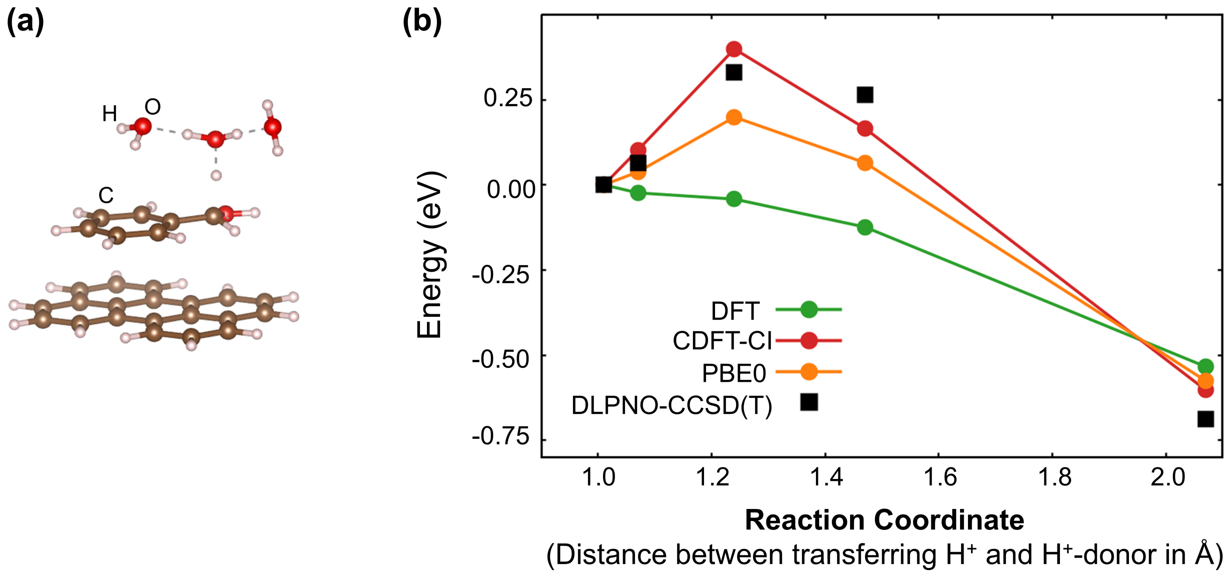

Figure S2. (a) A small cluster model for the validation test, where graphene is replaced with perylene ($C_{20}H_{12}$) while keeping the reactant and proton donor the same as in the main calculation. (b) The validation test for the CDFT-CI calculations was performed using DLPNO-CCSD(T) as a benchmark. The CDFT-CI calculations are consistent with the benchmark within 0.1 eV while DFT still underestimates the energies along the reaction coordinate. The results from PBE0 were also included. The results by all methods in (b) were obtained without implicit solvation. This is to facilitate an unbiased comparison for benchmarking purposes. In contrast, the CDFT-CI calculation in the main text includes implicit solvation.

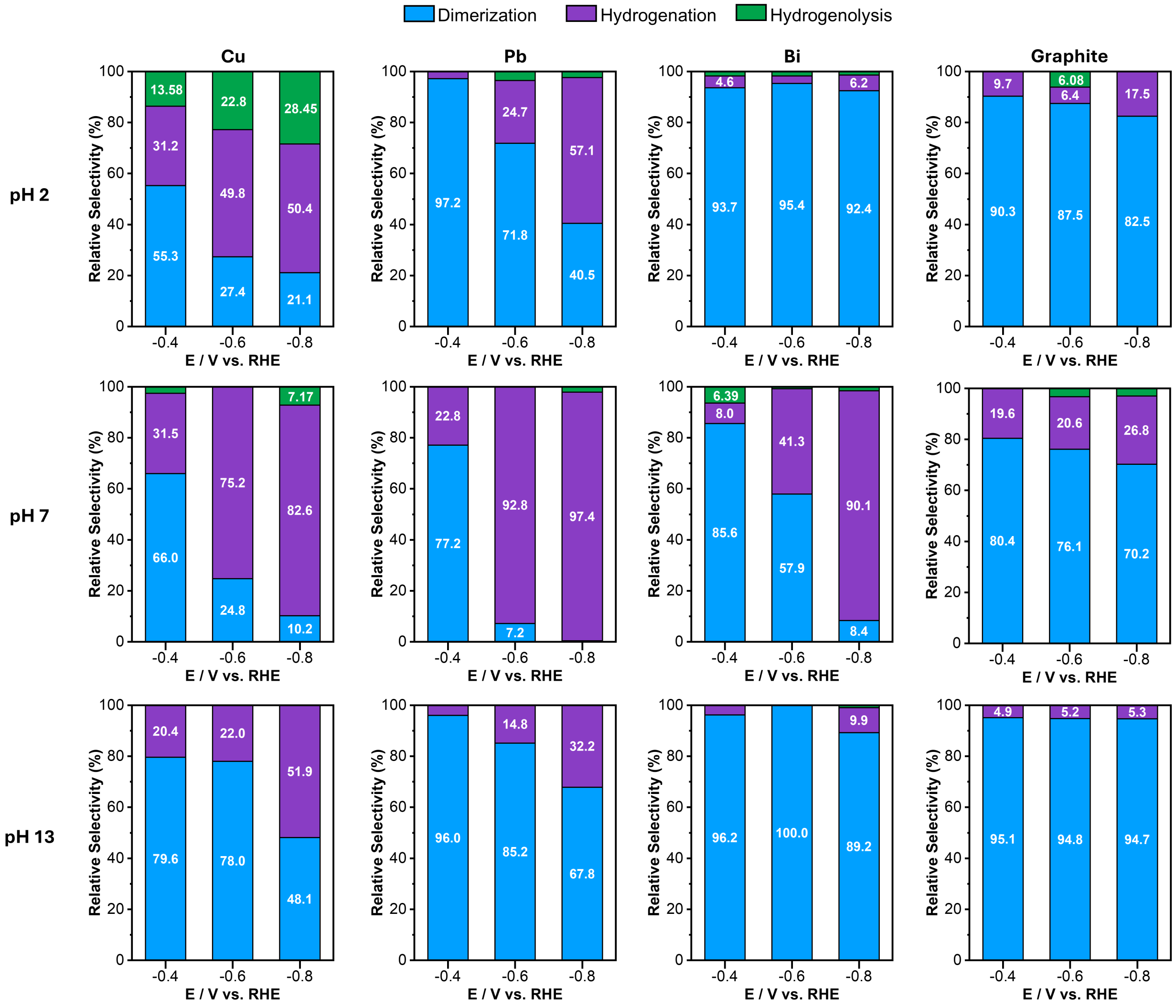

Figure S3. Relative selectivities (%) of identified products after passing 1 e^- per BAL molecule, using Cu, Pb, Bi, and graphite rod electrodes at pH 2, pH 7, and pH 13. Numbers on each bar show the numerical value of relative selectivity.

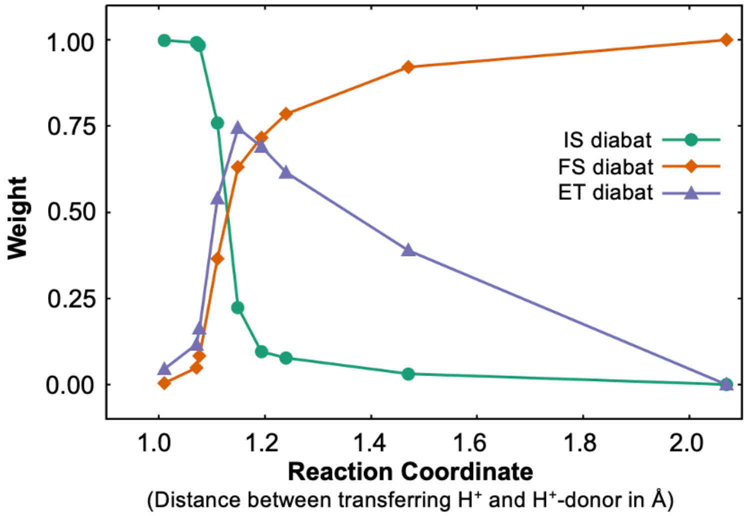

Figure S4. The distribution of weights in the CDFT-CI calculations as a function of the reaction coordinate. Note that the weights at the last configuration (the reaction coordinate of 2.1 Å) are 1 for the FS diabat and 0 for other diabats, which is justified by the fact that the CDFT calculation for the FS diabat already gives the electronic energy of the unconstrained DFT calculation within 0.025 eV.

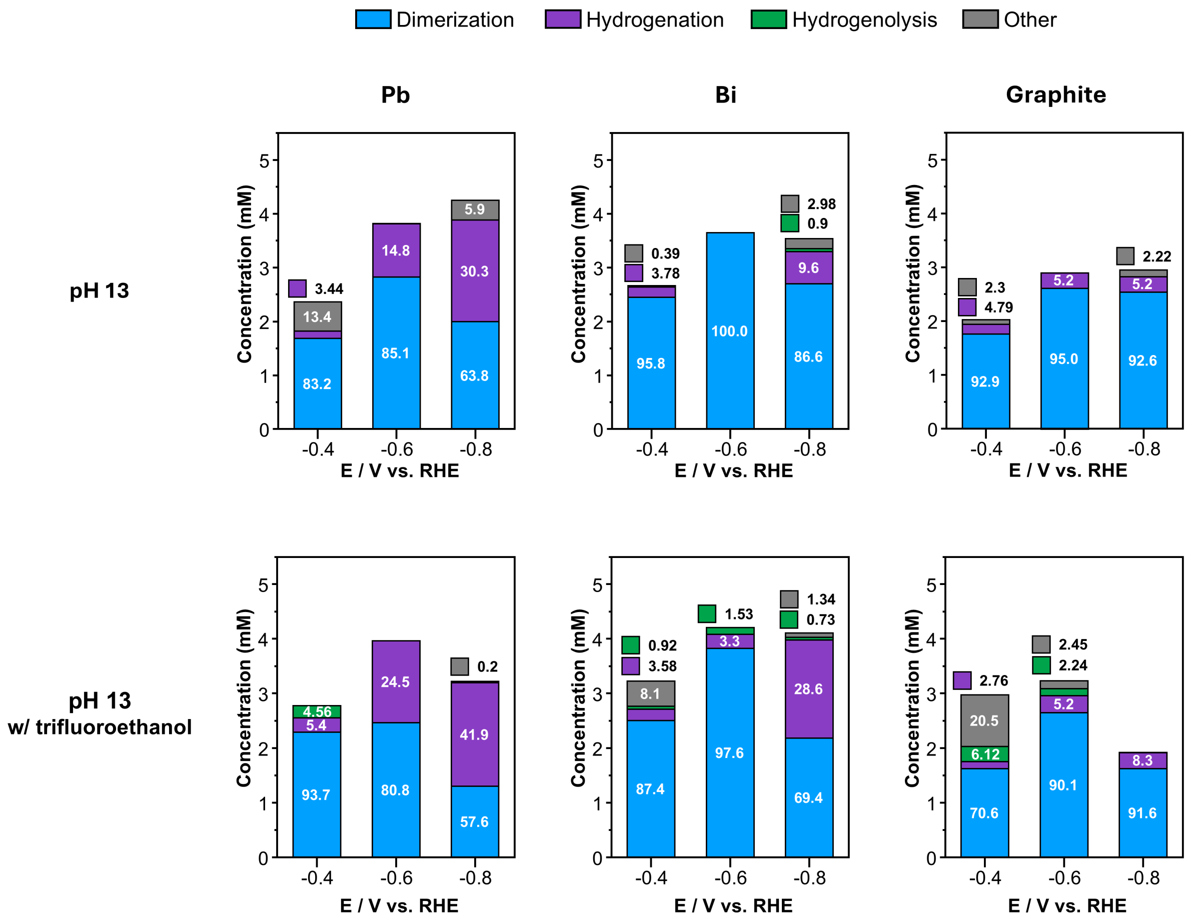

Figure S5. Comparison of product concentrations after passing 1 e⁻ per BAL molecule using Pb, Bi, and graphite rod electrodes at pH 13 without (top) and with (bottom) 0.7 M trifluoroethanol. Numbers on each bar show the absolute selectivity of each product. Almost all conditions show an increase in the hydrogenation selectivity with the presence of trifluoroethanol.

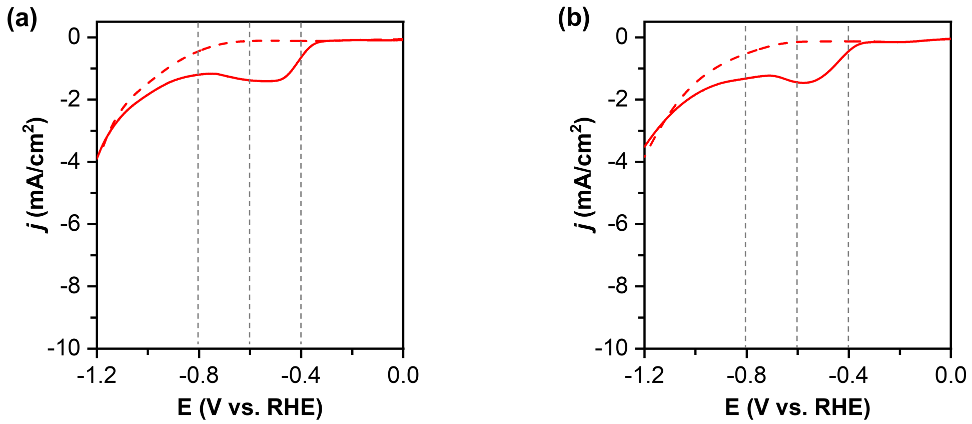

Figure S6. Comparison of LSVs of Bi at pH 13 (a) without and (b) with 0.7 M trifluoroethanol as an example showing the effect of trifluoroethanol on solution IR drop. The vertical dashed lines represent the potentials chosen for the constant potential BAL reduction. As the solution resistance of unbuffered pH 13 solution was already low, the addition of trifluoroethanol did not result in a notable difference in the solution IR drop, which was confirmed by the absence of notable changes in the reduction onset potential and current density in these LSVs.

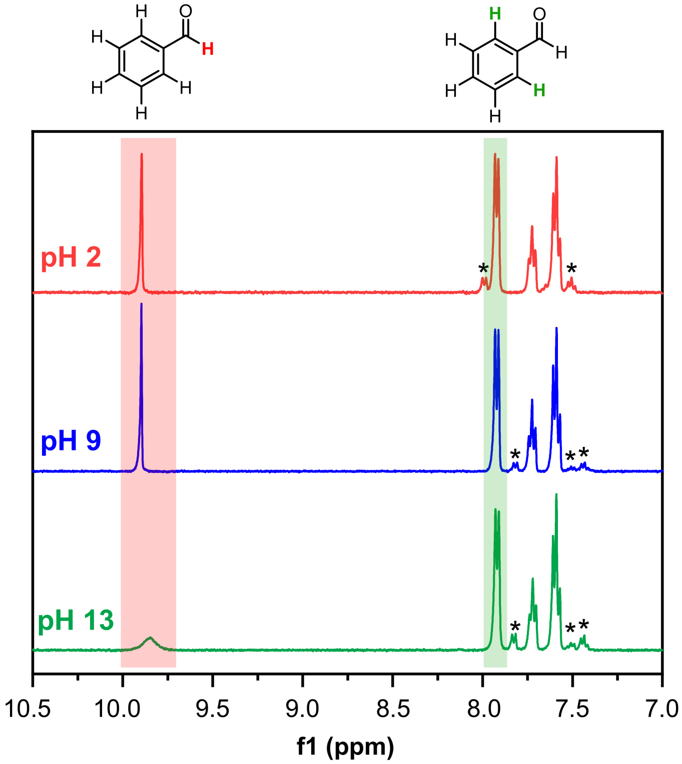

Figure S7. ^{1}H NMR spectra of 10 mM BAL in pH 2 (phosphate buffer), 9 (borate buffer), and 13 (0.1 M KOH). The decrease in the aldehyde hydrogen peak at pH 13 is due to the conversion of a fraction of BAL (~65%) to its geminal diol form (phenylmethanediol).[S1] The geminal diol peak is not shown as it lies in the water suppression region. The broadening and slight upfield shift of the aldehyde hydrogen peak at pH 13 indicates a fast equilibrium between aldehyde and geminal diol (i.e., occurring on the timescale of NMR). Peaks marked with "*" are due to benzoic acid present in our bottle of BAL as an impurity. Note that benzoic acid is present as benzoate at pH 9 and 13, shifting the peak positions.

Table S1. All numerical data used to make **Figures 3, 4, and S3.** Electrolysis time and average current for each electrolysis are also summarized.

pH 2

Electrode	Cu			Pb			Bi			graphite		
E / V vs. RHE	-0.6	-0.8	-1.0	-0.6	-0.8	-1.0	-0.6	-0.8	-1.0	-0.6	-0.8	-1.0
% conversion	40.69	19.04	16.06	49.59	65.48	57.2	37.03	66.68	52.55	30.84	57.57	39.3
% selectivity dimerization	32.63	18.26	9.49	84.99	65.4	36.81	76.39	80.17	76.88	35.77	62.46	52.17
% selectivity hydrogenation	18.42	33.26	22.66	2.48	22.44	51.94	3.72	2.44	5.14	3.82	4.59	11.07
% selectivity hydrogenolysis	8.02	15.22	12.78	0	3.22	2.19	1.46	1.45	1.18	0	4.34	0
% selectivity other	40.93	33.26	55.07	12.53	8.94	9.06	18.43	15.94	16.8	60.41	28.61	36.76
Time to pass 9.649 C / min	329.84	25.79	10.27	160.65	41.32	22.87	140.63	23.37	10.05	357.1	163.66	196.65
Ave. j / mA cm^{-2}	0.49	6.23	15.66	0.42	1.83	3.3	0.17	1.04	2.41	0.45	0.98	0.82

pH 7

Electrode	Cu			Pb			Bi			graphite		
E / V vs. RHE	-0.6	-0.8	-1.0	-0.6	-0.8	-1.0	-0.6	-0.8	-1.0	-0.6	-0.8	-1.0
% conversion	41.58	17.2	12.42	63.12	48.39	49.51	47.14	62.08	49.49	40.8	37.16	21.57
% selectivity dimerization	64.56	17.46	6.86	76.89	6.95	0.4	77.61	52.35	5.1	57.6	63.36	57.07
% selectivity hydrogenation	30.76	52.94	55.32	22.76	89.56	89.19	7.27	37.32	54.95	14.03	17.12	21.75
% selectivity hydrogenolysis	2.46	0	4.8	0	0	1.96	5.79	0.74	0.92	0	2.76	2.45
% selectivity other	2.22	29.6	33.02	0.35	3.49	8.45	9.33	9.59	39.03	28.37	16.76	18.73
Time to pass 9.649 C / min	86.3	13.48	5.73	56.31	19.05	17.08	112.23	24.6	6.84	140.2	36.97	33.5
Ave. j / mA cm^{-2}	1.86	11.93	28.06	1.01	2.97	3.31	0.22	0.98	3.54	1.15	4.35	4.8

pH 13

Electrode	Cu			Pb			Bi			graphite		
**E / V vs. RHE**	-0.6	-0.8	-1.0	-0.6	-0.8	-1.0	-0.6	-0.8	-1.0	-0.6	-0.8	-1.0
**% conversion**	37.41	34.65	14.55	42.28	69.34	65.33	53.48	76.22	65.29	38.65	53.89	53.8
**% selectivity dimerization**	74.75	70	37.18	83.19	85.14	63.84	95.83	100	86.55	92.91	95.01	92.56
**% selectivity hydrogenation**	19.12	19.74	40.14	3.44	14.83	30.28	3.78	0	9.57	4.79	5.23	5.22
**% selectivity hydrogenolysis**	0	0	0	0	0	0	0	0	0.9	0	0	0
**% selectivity other**	6.13	10.26	22.68	13.37	0.03	5.88	0.39	0	2.98	2.3	-0.24	2.22
**Time to pass 9.649 C / min**	159.28	25.88	8.45	104.8	25.66	41.99	78.5	23.64	14.41	65.35	36.64	33.72
**Ave. j / mA cm^{-2}**	1.01	6.21	19.04	0.66	2.7	1.65	0.31	1.02	1.68	2.46	4.39	4.77

Table S2. Adsorption energies (in eV) of CHO and CHOH on Cu, Pb, Bi, and graphite. Clean slabs of the electrodes and isolated CHO and CHOH are used as references. Values in parentheses are calculated with the density-dependent energy correction (dDsC).

	Cu	Pb	Bi	graphite
CHO	-1.05<br>(-0.68)	-0.40<br>(-0.41)	-0.32<br>(-0.33)	-0.54<br>(-0.59)
CHOH	-1.65<br>(-1.24)	-0.54<br>(-0.55)	-0.51<br>(-0.55)	-0.53<br>(-0.59)

References

(S1) Bender, M. T.; Warburton, R. E.; Hammes-Schiffer, S.; Choi, K. S. Understanding Hydrogen Atom and Hydride Transfer Processes during Electrochemical Alcohol and Aldehyde Oxidation. *ACS Catal* **2021**, *11*, 15110–15124.
